# Is human milk feeding protective for Retinopathy of Prematurity?

**DOI:** 10.12669/pjms.346.15799

**Published:** 2018

**Authors:** Ayesha Muneer, Attia Bari, Summaira Naveed, Agha Shabbir Ali

**Affiliations:** 1 *Dr. Ayesha Muneer, DCH, F.C.P.S. (Pediatric Medicine), Assistant Professor, Pediatric Medicine, The Lahore General Hospital, Lahore, Pakistan*; 2 *Dr. Attia Bari, MCPS, DCH, F.C.P.S. (Pediatric Medicine), MHPE. Associate Professor, Paediatric Medicine, The Children’s Hospital Lahore, Pakistan*; 3 *Dr. Summaira Naveed, F.C.P.S. (Pediatric Medicine), Assistant Professor, Pediatric Medicine, Sir Ganga Ram Hospital, Lahore, Pakistan*; 4 *Prof. Agha Shabbir Ali, MCPS, F.C.P.S. (Paediatric Medicine) Professor of Pediatric Medicine, The Lahore General Hospital, Lahore, Pakistan*

**Keywords:** Breast milk, Preterm, Retinopathy of prematurity

## Abstract

**Objective:**

To find the association between breast milk feeding with retinopathy of prematurity (ROP) in preterm infants.

**Methods:**

This was a cross sectional study to examine the effects of breast milk feeding on ROP. Premature newborns below 34 weeks from neonatal unit retinopathy of prematurity program during the years 2015 to 2017 of The Lahore General Hospital were included. We recorded the gestational age, birth weight, presence of ROP and the type of feeding (breastfeeding vs. formula milk).

**Results:**

Out of 428 preterm babies 210 (49%) were males. More babies were between 32-34 weeks of gestation 229 (53.5%) as compared to < 32 weeks 199 (46.5%). Among all 428 preterm infants 19(4.4%) developed ROP. Majority 13 (68.4%) who developed ROP were <32 weeks of gestation (*p=*0.042). The mean birth weight of infants without ROP was 1.51± 0.36 kg (95%CI; 1.47-1.55), while it was 1.36 ± 0.29 kg (95%CI; 1.22-1.50) with ROP and all who developed ROP were < 2kg. The estimated odds ratio of developing ROP for breast fed versus top feeding was (ORs: 0.571, 95% CI; 0.222- 1.489). There was a trend toward lower incidence of ROP in the group of newborns who received breast-feeding (36.8%) as compared to top feeding (63.2%) but almost similar percentage who didn’t develop ROP were breast fed or top fed with statistically insignificant results (p= 0.24).

**Conclusions:**

Slightly lesser percentage of preterm babies who were breast fed developed retinopathy of prematurity.

## INTRODUCTION

Advances in neonatal intensive care management of extremely premature infants in the developed world and now also in developing countries, has led to an increased survival and an increase risk of long term morbidities like retinopathy of prematurity (ROP).^1^ There are differing trends in the frequency and severity of ROP ranging from 10.5% to as high as 58%.^2^,^3^ In preterm neonates the development of any ROP is associated with both lower birth weight (BW) and lower gestational age (GA).^4^ There are various other risk factors for development of ROP which include neonatal sepsis, respiratory distress syndrome, anemia resulting in multiple blood transfusions and apnea of prematurity.^5^

ROP is considered as a preventable cause of blindness and therefore its screening is essential. Early screening and timely initiation of treatment of ROP is crucial in the prevention and treatment of this disease.^6^,^7^ If these babies are left untreated, they are at risk of developing vitreous hemorrhage, vitreoretinal fibrosis, and retinal detachment can develop in the most severe stages of the disease ultimately leading to permanent blindness.^5^

Prematurity related morbidities including sepsis and ROP are reduced in the incidence, severity and risk by effectiveness of human milk.^8^,^9^ The American Academy of Pediatrics recommends breast milk feedings for both preterm and-full term infants. A high concentration of long-chain polyunsaturated fatty acids is found in breast milk which is essential substrates for the developing brain and retina. Mother’s milk antioxidants protect the eye in preterm babies who are exposed to high oxygen concentrations during their admission in hospital and may minimize oxidative DNA damage.^10^,^11^ Breast feeding increase the odds of preventing ROP, which is the reason preterm babies can go blind.^12^

There was scarcity of research from Pakistan on ROP and there is not much data about the prevalence of ROP in newborn and its correlation with their gestational age and breast feeding. Due to the protective role of breast feeding in ROP and importance of screening in preterm babies we planned this study in our out-patient clinic, having strong follow up of premature babies discharged from our neonatal unit. The rationale of the study was to determine ROP in preterm babies and to find the association between ROP and breast feeding.

## METHODS

This prospective longitudinal study was conducted at The Lahore General Hospital. The study was approval by the Institutional Review and Ethics Board of the hospital. Written informed consent after describing purpose of data collection was taken from the parents of all children involved in the study. Sample size of 375 children was calculated using 95% confidence interval, 5% margin of error and taking 58% estimated prevalence of ROP.^3^ We had 428 preterm babies on our follow-up of ROP program so we extended our sample and it was more than the calculated as we included all babies in our study. All preterm newborns were included who were admitted at the neonatal intensive care unit and had a ROP screening between January 2015 and October 2017. All preterm babies without complete ROP screening outcomes and who died prior to ROP screening were excluded from the study.

Infants were initially screened using an indirect binocular ophthalmoscope at 15^th^ day of life and subsequently they had follow-up examination by ophthalmologist on weekly basis. Until retinal vasculature reached maturity, until any ROP regressed in severity, or until ROP required treatment. The severity or grading of ROP was done and recorded by ophthalmologist appropriately.^13^ Any baby who was suspected of requiring treatment was handed over to ophthalmologist for further treatment.

Data collected included GA, weight, gender, feeding practice (mother feed or formula/cow’s milk) till their final endpoint of retinal vascular maturity or development of ROP, presence and severity of ROP. GA was defined as the time between the first day of the last menstrual cycle and birth. International classification of ROP was used for classifying ROP. Data were statistically analyzed using Statistical Package for the Social Sciences (SPSS) version 20 software. Quantitative variables like BW were represented as mean and standard deviation and qualitative variables in frequency and percentages. Probability value of <0.05 was considered to be significant.

## RESULTS

Out of 428 preterm babies 210 (49%) were males. More babies were between 32-34 weeks of gestation 229 (53.5%) as compared to < 32 weeks 199 (46.5%) (Table-I). ROP was noted in 19 (4.4%). Majority 13 (68.4%) who developed ROP were <32 weeks of gestation (*p=*0.042) shown in (Table-II). The mean BW of infants without ROP was 1.51± 0.36 kg (95%CI; 1.47-1.55), while it was 1.36 ± 0.29 kg (95%CI; 1.22-1.50) with ROP. Maximum number of newborns had weight between 1-1.5 kg 196 (45.8%), 28 (6.5%) were <1kg and only 15 (3.5%) had weight > 2kg (Table-I). Almost equal percentage of newborns were breast fed 213 (49.8%) and top fed 215 (50.2%). The estimated odds ratio of developing ROP for breast fed versus top feeding was (ORs: 0.571, 95% CI; 0.222-1.489). There was a trend toward lower incidence of ROP in the group of newborns who received breast-feeding (36.8%) as compared to top feeding 12 (63.2%) but almost similar percentage who didn’t develop ROP were breast fed or top fed with statistically insignificant results (p= 0.24) (Table-II).

**Table-I:** Demographic characteristics of preterm babies.

Study Participants variables	Number (%)
***Gender***	
M:F	1:1
Male	210 (49)
Female	218 (51)
***Gestational age***	
<32 weeks	199 (46.5)
32-34 weeks	229 (53.5)
***Weight of baby***	
< 1 kg	28 (6.5)
1-1.5 kg	196 (45.8)
1.6- 2 kg	189 (44.2)
> 2 kg	15 (3.5)
***Type of feeding***	
Breast feed	213 (49.7)
Formula feed	157 (36.7)
Cow’s milk	58 (13.6)

## DISCUSSION

Increased survival of premature neonates as a result of advances in neonatal care has led to ultimately increase in long term morbidities like ROP. There is wide range of GA and BW in which ROP develop in premature neonates. In developed countries, far more preterm neonates are being saved as compared to developing world causing higher incidence of ROP in these extremely low BW and GA babies.

In our study the incidence of ROP was 4.4%. It is lower to the incidence reported in a study from Aga Khan University Hospital Pakistan,^2^ but very different from the studied in developed countries.^3^ This variation might be partly accounted by differences in the proportions of infants at high risk of ROP who survive when born at an early GA.^2^ ROP is often not recognized early because screening and treatment programs are not yet in place in most neonatal units, even in tertiary care hospitals. Studies from Bangladesh and Turkey showed a much higher incidence of 23.7% and 38.5% respectively.^3^,^14^

No association of ROP was found with gender of the baby as in our study almost similar percentage of premature neonates belonged to male and female. A Turkish study also showed similar findings with ROP occurring in both genders equally.^3^ Another study from Pakistan showed almost similar results with no association of ROP with gender (p 1.00).^2^

There is much higher incidence of ROP in neonates who are more premature and had much lower BW. Our study showed the low mean BW of babies who develop ROP [1.36 ± 0.29 kg (95% CI; 1.22-1.50)] as compared to premature babies who didn’t develop ROP [1.51±0.36 kg (95%CI; 1.47-1.55)]. Various studies from all over the world showed association of high incidence of ROP with low BW of prematurely born neonates. A study done by Sahin et al. showed that low BW and low GA was significantly associated with ROP (p 0.014 and 0.002) respectively, but in their study babies were significantly of lower GA (25.8±1.12 weeks) and lower BW (924.4 ± 205 gms).^3^ In our study, gestational age and birth weight were independently associated with ROP as 13 (68.4%) who develop ROP were <32 weeks’ gestation and that is consistent with many other studies.^2^,^7^,^14^ A study from china showed conflicting results about the association of birth weight with ROP as the birth weight of both groups with ROP or without ROP was similar with insignificant p-value.^6^

**Table-II:** Factor associated with retinopathy of prematurity.

Parameters	ROP n: 19 (4.4%)	No ROP n: 409 (95.6%)	p-value
***Gender***			
Male	10 (4.8)	200 (95.2)	0.466
Females	09 (4.1)	209 (95.9)	
***GA (weeks)***			
< 32	13 (6.5)	186 (93.5)	0.042
32- 34	06 (2.6)	223 (97.4)	
***Birth weight (kg)***			
<1.5 kg	14 (6.2)	210 (93.8)	0.046
>1.5 kg	05 (2.5)	199 (97.5)	
***Breast feeding***			
Breast milk	07 (3.3)	206 (96.7)	0.180
Top feed	12 (5.6)	203 (94.4)	

Multiple studies have been published to determine the association of breast feeding with ROP and the results reported are conflicting. In our study, comparatively more children who didn’t develop ROP were breast fed as compared to those who developed ROP although results were not statistically significant. A study from US by Cherri et al. reported that in extremely low birth weight babies human milk intake was associated with decreased risk of developing ROP.^15^ Ginovart from Spain reported that breast feeding reduced the risk for any stage of ROP by 75% in preterm infants (OR=0.25, 95% CI; 0.091-0.705) (p 0.009).^16^ A similar study published in Journal of Perinatology also showed that incidence of ROP differ significantly by type of feed given to preterm baby and human milk provision is correlated with reduced odds of ROP (OR:0.42, 95% CI: 0.19- 0.93, p= 0.003).^17^ A meta-analysis published by Zhuo also supported the protective role of human milk feeding in preventing any stage of ROP.^18^ In all those preterm newborns who developed ROP in our study, we found breast feeding to be associated with lesser risk of developing ROP as compared to those babies who were top fed, but almost similar percentage who didn’t develop ROP were breast fed or top fed with statistically insignificant results (p=0.24). A study published in Arch Pediatr Adolesc Med showed opposite result and revealed that mother’s milk has a role only in decreasing neonatal sepsis but it does not affect other morbidities like ROP in low BW babies.^9^ Due to these varied results, more studies are needed to investigate the impact of breast feeding on ROP.

### Limitation

ROP is significantly associated with prolonged oxygen therapy and these premature babies often require oxygen for a longer duration. The need and duration of oxygen therapy was not taken into account in our study.

## CONCLUSION

In our study the incidence of ROP was amongst the lowest reported. Our findings suggest that, preterm babies born at <32 weeks GA, <1.5 kg BW and babies who are on top feeding other than human milk feeding are at risk for ROP development. Our findings provide important clinical insight for follow-up of preterm babies to minimize long-term visual morbidity although further studies are required.

### Authors’ Contribution

**AM:** Conceived idea, data collection.

**AB:** Main author, manuscript writing.

**SN:** Critical review, final drafting.

**ASA:** Suggestions, final Approval.
